# Intraspecific Variation in the Characteristics of *Cryptocaryon irritans* Isolated in Japan

**DOI:** 10.1111/jfd.70177

**Published:** 2026-04-01

**Authors:** Hiromi Matsuoka, Toshihide Iwatsuki, Maho Kotake, Tomoki Honryo, Sho Shirakashi, Naoki Itoh, Yuho Watanabe

**Affiliations:** ^1^ Department of Aquatic Bioscience, Graduate School of Agricultural and Life Sciences The University of Tokyo Tokyo Japan; ^2^ Aquaculture Research Institute Kindai University Higashimuro Japan; ^3^ Aquaculture Research Institute Kindai University Nishimuro Japan

**Keywords:** biological diversity, coastal Japan, *Cryptocaryon irritans*, cryptocaryoniasis, intraspecific variation, salinity and temperature

## Abstract

*Cryptocaryon irritans* is an obligate parasitic ciliate and the causative agent of cryptocaryoniasis. This parasite is widely distributed in tropical and subtropical marine waters, and numerous outbreaks in aquaculture have been reported worldwide. Previous studies have documented variations in biological traits, such as cell size, serotype and environmental conditions for infection, including salinity and temperature, suggesting considerable intraspecific diversity. Molecular and serological variations have been reported in Japan; however, the extent of biological diversity remains unclear. Understanding this variation is critical because it may influence experimental outcomes and control strategies. In this study, *C. irritans* was collected from three geographically proximate coastal sites in Japan, and clonal cultures were established. Biological characteristics—including cell size, serotype, pathogenicity and development and infectivity under different salinity and temperature conditions—were compared to assess intraspecific variation. Genetic diversity was also evaluated using phylogenetic analysis. Biological characteristics differed even among isolates from nearby sites, indicating the presence of biologically distinct populations within Japan. Moreover, phenotypic comparisons and phylogenetic analyses showed that distinct traits were observed not only between genetically distant isolates but also within the same group, underscoring high intraspecific diversity. These findings may contribute to guiding future research and developing effective control strategies for cryptocaryoniasis.

## 1 Introduction

*Cryptocaryon irritans* (Brown 1951) is an obligate parasitic ciliate that infects various marine teleosts and causes cryptocaryoniasis or ‘marine white spot disease’. The parasite invades the epithelial layer of the skin and gills of fish and perturbs the osmotic control and respiratory activity of its host. Heavy infections often lead to mass mortality in affected fish (Colorni and Burgess 1997).

The life cycle of *C. irritans* comprises five developmental stages: theront, trophont, protomont, tomont and tomite (Sikama 1937; Brown 1963; Wilkie and Gordin 1969; Colorni 1985, 1987). Theront is a free‐swimming and invasive stage, wherein *C. irritans* enters the fish's surface tissues (including the skin, fins and gills) and transforms into the trophont, which is its parasitic stage. The trophont feeds and grows on these surface tissues, where it matures and eventually leaves the host as a protomont. The protomont then sinks, settles on the substrate and encysts as a tomont. Tomont undergoes cell division, via which daughter cells or ‘tomites’ are produced. Completely developed tomites are released into seawater as theronts (Brown 1963; Colorni 1985, 1987; Dickerson 2006; Nigrelli and Ruggieri 1966; Wilkie and Gordin 1969).

*C. irritans* is widely distributed in warm marine waters worldwide and has been the subject of extensive research because of its significant impact on marine aquaculture. Previous studies have revealed considerable variations in the biological characteristics of isolates from different geographic regions (Li et al. 2022). For example, Diggles and Lester (1996a) compared two isolates collected from Moreton Bay and Heron Island in Australia and found significant differences in the parasite size and developmental rate. Similarly, Yambot et al. (2003) reported isolate‐dependent variation in these traits among parasites collected from different locations in Taiwan. In Hawaii, Misumi et al. (2011) observed serological differences between the two isolates. Previous studies have documented that cryptocaryoniasis outbreaks can occur under widely differing environmental conditions, including salinity and temperature (Diggles and Lester 1996a; Diggles and Adlard 1997; Yambot et al. 2003). These findings suggest the presence of diverse *C. irritans* isolates with distinct biological characteristics worldwide.

However, in Japan, research on *C. irritans* has largely focused on the development of chemotherapeutic treatments, vaccines and detection methods (Yoshinaga et al. 2011; Kawano et al. 2012; Watanabe et al. 2021, 2022; Taniguchi et al. 2011; Imajoh et al. 2016), reflecting the lack of effective control strategies for cryptocaryoniasis. Consequently, little attention has been paid to the biological characteristics or intraspecific diversity of these parasites. Although genetically distinct and serologically different isolates have been reported (Hatanaka et al. 2007; Imajoh et al. 2016), information on their biological traits remains limited, and it is still unclear whether biologically distinct *C. irritans* isolates exist in Japanese waters. Clarifying the biological diversity of *C. irritans* in domestic marine environments could have important implications for future research and the development of effective control measures.

In this study, we collected *C. irritans* samples from three adjacent marine locations in Japan, established clonal isolates from each site, and compared their biological characteristics to assess whether phenotypic diversity exists among the isolates inhabiting geographically proximate regions.

## 2 Materials and Methods

### 2.1 Fish

Black molly (*Poecilia* spp.) and olive flounder ( *Paralichthys olivaceus* ) were used for parasitic maintenance and/or experimental infection.

Black mollies (total length, 2–3 cm) were purchased from Japan Animal Medicine Co. Ltd. They were reared in freshwater and had no history of infection with *C. irritans* . Until use, the fish were maintained in a 500‐L tank equipped with a filtration system under the following conditions: 23°C, salinity 20‰, pH 7.0–7.5 and dissolved oxygen above 98% saturation. During the maintenance period, fish were fed to satiation once daily with a commercial diet (Otohime C1; Nisshin Marubeni Feed Co. Ltd., Tokyo, Japan).

Japanese flounder (total length, 6.0–7.0 cm) were purchased from Marintech Co. Ltd. and maintained under similar conditions in a 500‐L filtered tank at 20°C, salinity 30‰, pH 7.0–7.5 and oxygen saturation above 98% until use. The fish were fed a commercial diet (Otohime EP1; Nisshin Marubeni Feed Co. Ltd.) once daily for satiation. Our laboratory has been purchasing marine fish from this aquaculture facility for over 20 years, and no infection with *C. irritans* has been detected in the fish obtained from this source. Upon arrival, five fish were sampled and confirmed to be free of *C. irritans* .

### 2.2 Parasite

#### 2.2.1 Collection and Propagation of *C. irritans*

**FIGURE 1:**
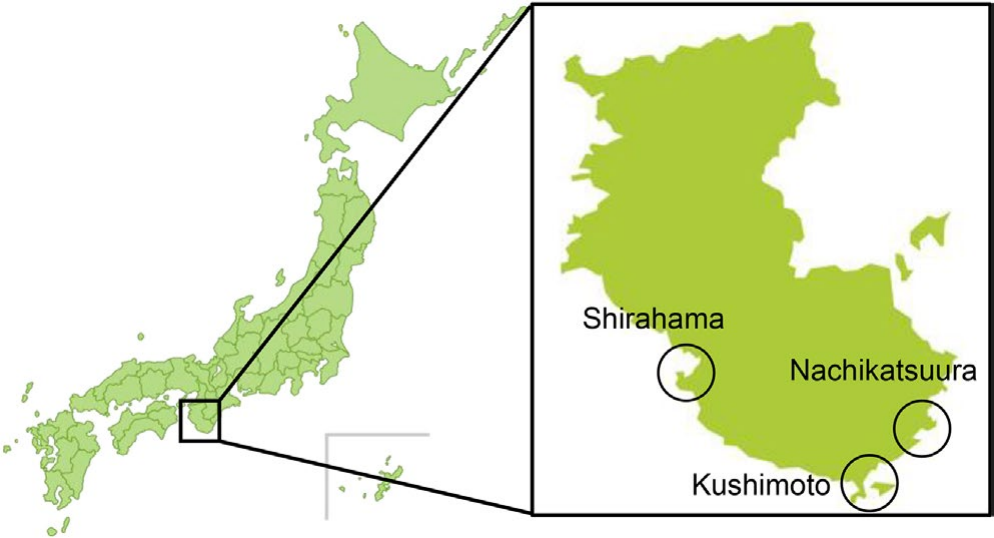
Sampling sites of fish specimens infected by *Cryptocaryon irritans*.

The isolates of *C. irritans* used in this study were obtained from infected fish collected from three locations in Wakayama Prefecture: Kushimoto, Shirahama and Nachikatsuura (Figure 1). Details of the collection period, host fish species and origin of each isolate are shown in Table 1. Each isolate was passaged and propagated on seawater‐adapted black mollies as described by Watanabe et al. (2020).

**TABLE 1:** Information of three samples of *Cryptocaryon irritans*.

Samples	Locations	Dates	Host	Sources
WK‐1	Wakayama Pref. Kushimoto	Jun. 2023	*Sebastiscus marmoratus*	Wild‐caught
WS‐1	Wakayama Pref. Shirahama	May 2023	*Sebastiscus marmoratus*	Wild‐caught
WN‐1	Wakayama Pref. Nachikatsuura	Aug. 2023	*Pagrus major*	Offshore fish farm

#### 2.2.2 Establishment of Clonal Isolates

Clonal isolates of *C. irritans* derived from Kushimoto, Shirahama and Nachikatsuura were established as follows:

Protomonts collected during the serial propagation passages were transferred using a Pasteur pipette into a glass bowl (outer diameter: 10 cm, depth: 4 cm; Stack Bowl, Arcoroc Co., France) containing 50 mL of artificial seawater (28°C, 30‰) supplemented with antibiotics (500 IU/mL penicillin G potassium and 500 μg/mL streptomycin sulfate). The parasites were transferred to another bowl containing antibiotic‐supplemented artificial seawater under a stereomicroscope (SZ61; Olympus Co., Tokyo, Japan). This washing step was repeated thrice to ensure the removal of debris and contaminants. After washing, individual protomonts were transferred into separate wells of a 96‐well plate (AS ONE Co., Osaka, Japan), each containing 100 μL of artificial seawater, and incubated at 28°C for 3 h to allow to transform into tomonts. The tomonts were then incubated at 28°C until their release was confirmed. Once theronts were observed, all seawater from the well was transferred into a 300 mL beaker (AS ONE Co.) containing one black molly and 100 mL of artificial seawater (28°C, 30‰) to initiate infection. Following infection, the fish were maintained according to the method described by Watanabe et al. (2020). Parasites detached from the infected fish were incubated, and theronts released from the tomonts were used to infect new fish. This procedure was repeated to establish clonal isolates of *C. irritans* .

Three clonal isolates established using this procedure were used in the subsequent experiments. The isolates derived from Kushimoto, Shirahama and Nachikatsuura were designated WK‐1, WS‐1 and WN‐1, respectively.

### 2.3 Phylogenetic Analysis

To characterise the genetic differences among the isolates, DNA was extracted from approximately 1000 protomonts using the QIAamp DNA Mini Kit (Qiagen, Valencia, CA, USA). Polymerase chain reaction (PCR) amplification was performed targeting two genetic regions, the mitochondrial cytochrome c oxidase subunit 1 (cox‐1) gene (Chi et al. 2017), and the internal transcribed spacer region 1, including partial 18S rRNA, ITS‐1, 5.8S rRNA and partial ITS‐2 sequences (Xie et al. 2021; Chen et al. 2008)—using the primer sets cox‐1F/R and Cryp‐F/S15 (Table 2). The PCR conditions used are as follows: 40 cycles of denaturation at 98°C for 10 s, annealing at 55°C for 5 s and extension at 68°C for 5 s. Each amplified product was cloned into the pGEM‐T Easy Vector (Promega, Madison, WI, USA), and sequenced by Eurofins Genomics K.K. (Tokyo, Japan). The obtained sequences were genotyped using phylogenetic analysis of previously reported *C. irritans* isolates (Supporting Information Data S1, Tables S1 and S2).

**TABLE 2:** Primers used in this study.

Primers		Sequence 5′–3′	References
Cox‐1	F	CAGAAGCCATACCTTGTCACCCTGTAA	Chi et al. (2017)
	R	TCTATGTGAACTGGAATGTCAGGTGCA	
18S‐ITS1	Cryp‐F	CACTAGTTAGTGCGGGAAGT	Xie et al. (2021)
	S15	TGAGAGAATTAATCATAATTTATAT	Chen et al. (2008)

To construct the phylogenetic trees, the cox‐1 sequences of *C. irritans* were retrieved from the GenBank database (Table S1), aligned using default settings and pairwise distances were calculated using MEGA version 12 (Kumar et al. 2024). The best‐fit substitution model for Maximum Likelihood (ML) analysis was determined using the model selection function implemented in MEGA12, and the GTR + G model, selected using the AICc criterion, was applied for ML tree construction. The robustness of the resulting topology was assessed using 1000 bootstrap replicates.

Phylogenetic analysis was conducted using the ITS1 region of ribosomal RNA from *C. irritans* isolates reported in previous studies (Table S2), following Chi et al. (2017). The ML trees were constructed as described above, except that the T92 + I model was used.

### 2.4 Comparison of Size of Each Isolate

#### 2.4.1 Protomonts

For each isolate, 30 protomonts obtained during the propagation process were individually transferred into the wells of a 96‐well plate, each containing 100 μL of artificial seawater (28°C, 30‰). To fix the parasites, 10 μL of 10% formalin was added to each well. The protomonts were imaged under an inverted microscope using image analysis software (cellSens Standard, Olympus Co.), and their body length (major axis) was measured.

#### 2.4.2 Tomonts

Thirty protomonts were placed individually into the wells of a 96‐well plate containing 100 μL of artificial seawater (28°C, 30‰) and incubated at 28°C for 3 h to induce encystment. The resulting tomonts were incubated for 24 h. After this period, 10 μL 10% formalin was added to each well for fixation. The tomonts were imaged and their body lengths were measured.

#### 2.4.3 Theronts

For theronts, 30 protomonts were placed in a Petri dish (φ40 × 13.5 mm; AS ONE Co.) containing 3 mL artificial seawater (28°C, 30‰), and incubated for 3 h to allow encystment. The tomonts were then incubated at 28°C for 4 days. During the incubation period, approximately 80% of the seawater was replaced every 2 days. On Day 4, theronts released from tomonts were collected and transferred into the wells of a 96‐well plate along with 50 μL seawater. Each sample was fixed by adding 10 μL 10% formalin. From each sample, 30 theronts were randomly selected and imaged, and their body lengths were measured.

These measurements were performed using protomonts collected from the three isolates on the same day during propagation using black mollies under identical conditions. In addition, the measurements were repeated using protomonts detached from the host on different days following the same procedure.

### 2.5 Number of Theronts Released From a Tomont

Protomonts were transferred into the wells of a 96‐well plate, each containing 100 μL artificial seawater (28°C, 30‰), and incubated at 28°C for 3 h to allow encystment. The resulting tomonts were maintained under the same conditions for 4 days. During this period, 80% of the seawater in each well was replaced every 2 days. On Day 4 after protomont collection, theront release was confirmed, and each well was fixed by adding 10 μL 10% formalin. The theronts released from each tomont were counted using an inverted microscope. Thirty protomonts from each isolate, collected on the same day, were used for the measurements. The experiment was repeated using protomonts collected on different days, following the same procedure.

### 2.6 Comparison of Serotypes

#### 2.6.1 Preparation of Antisera Against Each Isolate

To compare the serotypes of the three isolates, antisera specific to each isolate were prepared following the method described by Yoshinaga and Nakazoe (1997), with slight modifications. Eighteen uninfected olive flounders and six 3‐L plastic tanks were prepared. Each tank was filled with 1 L artificial seawater (25°C, 30‰), and three fish were placed in each. The six tanks were divided into three groups corresponding to the WK‐1, WS‐1 and WN‐1 isolates. Theronts (6000 cells per tank; 2000 cells per fish) of the respective isolates were added to each tank to initiate infection. Six hours after exposure, 1 L additional artificial seawater (25°C, 30‰) was added to each tank, bringing the total volume to 2 L. Infected fish were maintained at 25°C under aeration, and approximately 80% of the water was replaced daily. Two days after infection, trophonts on the fish surface were visually confirmed. The fish were then transferred to new 3‐L tanks containing 2 L of artificial seawater and held overnight. Fish were transferred daily to freshly prepared tanks and protomont detachment was monitored. Six days after infection, no further detachment of the protomonts was observed. Each infection‐experienced fish was transferred to a 60‐cm glass aquarium (600 × 300 × 360 mm) equipped with a filtration system and maintained for 4 weeks (25°C, 30‰, pH 7.0–7.5, > 98% dissolved oxygen). During this period, the fish were fed a commercial diet once daily until satiation. At the end of the 4‐week period, the fish were anaesthetised in 2 L of artificial seawater containing 400 μL of 2‐phenoxyethanol (Fujifilm Wako Pure Chemical Co., Osaka, Japan). Blood was collected from the caudal vein using a syringe and transferred into 1.5 mL tubes. After standing overnight at 4°C, the serum was separated via centrifugation and heat‐inactivated at 56°C for 30 min. The resulting antisera were used for subsequent serological tests. The protein concentrations of the antisera were measured using the TaKaRa BCA Protein Assay Kit (Takara Bio Inc., Shiga, Japan).

#### 2.6.2 Serotype Comparison Using Immobilisation and Agglutination Assay

To determine the serotype of each isolate, immobilisation and agglutination assays were performed using the corresponding antisera, following the method described by Yoshinaga and Nakazoe (1997), with slight modifications. Each antiserum sample was diluted with artificial seawater (30‰) to obtain a protein concentration of 5 mg/mL. Twofold serial dilutions were prepared from this stock to obtain final dilutions of 1:10–1:160. Theronts of each isolate were collected during the propagation process and adjusted to a density of 2000 cells/mL using artificial seawater. In each well of a 96‐well plate, 50 μL diluted antiserum and 50 μL theront suspension (final concentration, 100 cells/well) were mixed and incubated at 25°C for 1 h. After incubation, the theronts were examined under an inverted microscope for immobilisation and agglutination. The highest dilution at which a positive immobilisation reaction was observed was recorded as the immobilisation titre. Samples showing no immobilisation reactions, even at the lowest dilution (1:20), were considered negative.

### 2.7 Comparison of Pathogenicity

#### 2.7.1 Comparison of Pathogenicity Among the Three Isolates

To evaluate the pathogenicity of each isolate, infection trials were conducted by using a modified version of the method described by Watanabe et al. (2019). Forty‐eight black mollies were individually placed in 300 mL plastic beakers containing 300 mL of artificial seawater (25°C, 30‰) and acclimated for 7 days under the following conditions: 25°C, salinity 30‰, pH 7.0–7.5 and > 80% oxygen saturation. After acclimation, the fish were randomly assigned to four groups (*n* = 12 each): three experimental groups (challenged with WK‐1, WS‐1 or WN‐1) and one negative control group. Fresh theronts of each isolate were collected within 1 h of excystment and adjusted to a concentration of 1000 cells/mL. For infection, the water volume in each beaker was reduced to 100 mL and 1000 theronts were added to each beaker. In the negative control group, 1 mL of artificial seawater was added. After exposure, the beakers were left undisturbed for 6 h, after which 200 mL of artificial seawater was added to each beaker, and the fish were maintained at 25°C. Daily water changes (200 mL) were performed, and fish survival was recorded daily for 7 days post‐infection.

The infectivity of the theronts was assessed in parallel to the aforementioned experiment. Another set of 48 black mollies acclimated to the same conditions was randomly divided into four groups (*n* = 12 each). Fresh theronts of each isolate were collected, adjusted to 1000 cells/mL, and 100 theronts were added to each beaker. The negative control group received 100 μL of artificial seawater. The fish were maintained following the protocol described above, with 200 mL of seawater added 6 h post‐infection and replaced daily. On Day 3 post‐infection, mesh baskets were placed in the beakers from Day 2 onwards to collect the detached parasites. The number of protomonts and tomonts that settled at the bottom of each beaker was counted daily, and the cumulative number of parasites recovered by Day 7 was used to calculate the infection success per fish. The infectivity was defined as the number of parasites recovered per fish divided by the initial inoculum (100 theronts).

Food was not provided during the experimental period. Each experiment was repeated twice using theronts collected on different days. At the time of the first trial, the isolates were maintained in culture for 5 (WK‐1), 6 (WS‐1) and 3 months (WN‐1). The duration of the second trial was 7, 8 and 5 months, respectively.

#### 2.7.2 Effect of Serial Passage Duration on Pathogenicity

To examine the effect of passage duration on pathogenicity, an additional challenge experiment was conducted using a newly established clonal isolate (WS‐2, Table S3). WS‐2 was isolated in March 2024 from a mottled spinefoot ( *Siganus fuscescens* ) reared at the same aquaculture facility as the WS‐1 isolate and established as a clonal isolate following the procedures described in Section 2.2.2.

At the time of the experiment, the WN‐1 isolate had been maintained in culture for 10 months, whereas the WS‐2 isolate had been maintained for 2 months. Challenge experiments were conducted as described in Section 2.7.1. Pathogenicity was evaluated based on the survival of black mollies for 7 days post‐infection. Infectivity was assessed in parallel using the same protocol as described above.

### 2.8 Effects of Salinity on Each Isolate

To determine the effects of salinity, we modified the method described by Kong et al. (2022) and examined cyst formation in protomonts, theront release from tomonts and theront infectivity at various salinities. Owing to space constraints, the experiments were conducted separately for each isolate on different dates.

#### 2.8.1 Cyst Formation of Protomonts

Three Petri dishes (40 × 13 mm; AS ONE Co.) containing 3 mL artificial seawater at each of eight salinities (5, 10, 15, 20, 25, 30, 35 and 40) were prepared. Thirty protomonts collected from infected black mollies were added to each dish and incubated at 28°C for 24 h. The number of protomont‐forming cysts was counted under a stereomicroscope, and the cyst formation rate was calculated as the proportion of encysted individuals.

#### 2.8.2 Theront Release From Tomonts

Tomonts formed in the above experiment (Section 2.8.1) were maintained at 28°C for 6 days. The number of tomonts that released theronts was recorded daily until no further release occurred. The total number of theront‐releasing tomonts within 7 days was used to calculate the theront release rate based on the initial 30 individuals per dish. Water was changed every 2 days, and daily for dishes with theront release.

#### 2.8.3 Theront Infectivity

To assess infectivity, 70 black mollies were individually placed in 300 mL beakers and divided into seven groups (*n* = 10), each acclimated for 7 days at a different salinity (10‰–40‰) at 25°C (pH 7.0–7.5, > 80% oxygen). Theronts released 5 days after protomont collection were adjusted to 1000 cells/mL. For infection, the seawater in each beaker was reduced to 100 mL and 100 theronts from the corresponding salinity conditions were added. After 6 h, 200 mL of fresh seawater was added and the fish were maintained for 6 days with daily water changes. On Day 2, detached parasites were collected and counted daily. Infectivity of the theronts was calculated as the number of recovered parasites per fish divided by the number of theronts administered. Feeding was not performed during the experimental period.

The entire experimental series was repeated twice using parasites collected on different dates.

### 2.9 Effects of Temperature on Each Isolate

To evaluate the effects of temperature on each isolate, protomont cyst formation, theront release from tomonts and theront infectivity were examined under different temperature conditions, according to Yoshinaga (2001) with slight modifications.

#### 2.9.1 Cyst Formation of Protomonts at Different Temperatures

Fifteen petri dishes (40 × 13 mm) were prepared, each containing 3 mL artificial seawater (30‰) and 30 protomonts collected from infected black molly. Dishes were placed in incubators set at five temperatures (15°C, 20°C, 25°C, 30°C and 35°C), with three replicates per condition, and incubated for 24 h. The number of protomont‐forming cysts was counted under a stereomicroscope, and the cyst formation rate was calculated as the proportion of encysted individuals.

#### 2.9.2 Theront Release From Tomonts at Different Temperatures

Tomonts from the above experiment (Section 2.8.2) were further incubated at the respective temperatures (15°C–35°C) for 13 days. Theront‐releasing tomonts were counted daily until no further release was observed. During the experiment, 80% of the water was exchanged every 2 days using pre‐warmed artificial seawater; for dishes where theront release occurred, the water was changed daily. The theront release rate was calculated as the proportion of tomonts that released theronts within 13 days relative to the initial number (30 tomonts per dish).

#### 2.9.3 Infectivity of Theronts at Different Temperatures

Theront infectivity was assessed via a challenge experiment using black mollies. Owing to temperature‐dependent variation in the theront‐release timing, infection experiments were conducted on different days for each temperature condition. Seven days prior to the challenge, the black mollies were acclimated to the target temperatures. For each condition, 10 fish were individually placed in 300 mL beakers containing artificial seawater and maintained at their respective temperatures for 7 days. Preliminary experiments showed that theront release occurred at different times post‐cyst formation depending on temperature: 4 days at 30°C and 35°C, 5 days at 25°C, 7 days at 20°C and 8 days at 15°C. Based on these timings, acclimation began 7 days before the expected theront release for each condition. Water was exchanged daily (80%) with pre‐warmed artificial seawater. Theronts released under each temperature condition were collected in 15 mL tubes and adjusted to a concentration of 1000 cells/mL. On the day of infection, the volume of water in each beaker was reduced to 100 mL. Subsequently, 100 theronts collected within 1 h of excystment were added to each beaker. After 6‐h exposure, 200 mL artificial seawater pre‐adjusted to the target temperature was added, and the fish were maintained at that temperature for 6 days with daily water changes. From Day 2 post‐infection, the protomonts and tomonts detached from the host were counted. For each fish, the total number of parasites recovered until detachment ceased was recorded as the infection count. The infection rate was calculated as the number of recovered parasites per fish divided by the number of therapeutics administered. Feeding was not performed during the experiment.

All procedures were conducted separately for each isolate and were repeated twice using parasites obtained on different dates.

### 2.10 Statistical Analysis

Statistical analyses were performed using R software (version 4.5.2; R Foundation for Statistical Computing, Vienna, Austria) and RStudio (version 2026.01.0 + 329; Posit Software, PBC, Boston, MA, USA). Normality was assessed by visual inspection of histograms and Q–Q plots, supplemented by the Shapiro–Wilk test. Homoscedasticity was evaluated visually using residual plots and statistically with Levene's test.

Differences in major axis length at each developmental stage and in the number of theronts released from a single tomont were analysed using the Kruskal–Wallis test, followed by the Steel–Dwass multiple comparison test.

The infection rate per fish in the pathogenicity assessment was analysed in the same manner, as normality and homoscedasticity were not satisfied in some groups based on both visual inspection and statistical tests, and the parasite count data were overdispersed and deviated from normality (Crofton 1971). The effect of passage number on infectivity was evaluated using the Mann–Whitney U test for two‐group comparisons. Survival curves of black mollies were compared using the log‐rank test, with *p*‐values adjusted by the Bonferroni correction for pairwise comparisons.

For salinity and water temperature experiments, the proportions of trophonts that formed cysts and those that released theronts were arcsine square root transformed prior to analysis. Although normality was not satisfied in a few groups, it was met in most groups and homoscedasticity was confirmed for all groups; therefore, the data were analyzed using one‐way analysis of variance (ANOVA) followed by the Tukey–Kramer multiple comparison test. For the challenge test, the infection rate per fish within each isolate was analyzed using the Kruskal–Wallis test followed by the Steel–Dwass test.

Statistical significance was set at *p* < 0.05.

### 2.11 Ethics Statement

Although fish are not covered by national or university animal ethics protocols, the experimental fish were handled in a manner that minimised unnecessary pain. To this end, 2‐phenoxyethanol was used for anaesthesia and euthanasia in accordance with ethical considerations.

## 3 Results

### 3.1 Phylogenetic Analysis

**FIGURE 2:**
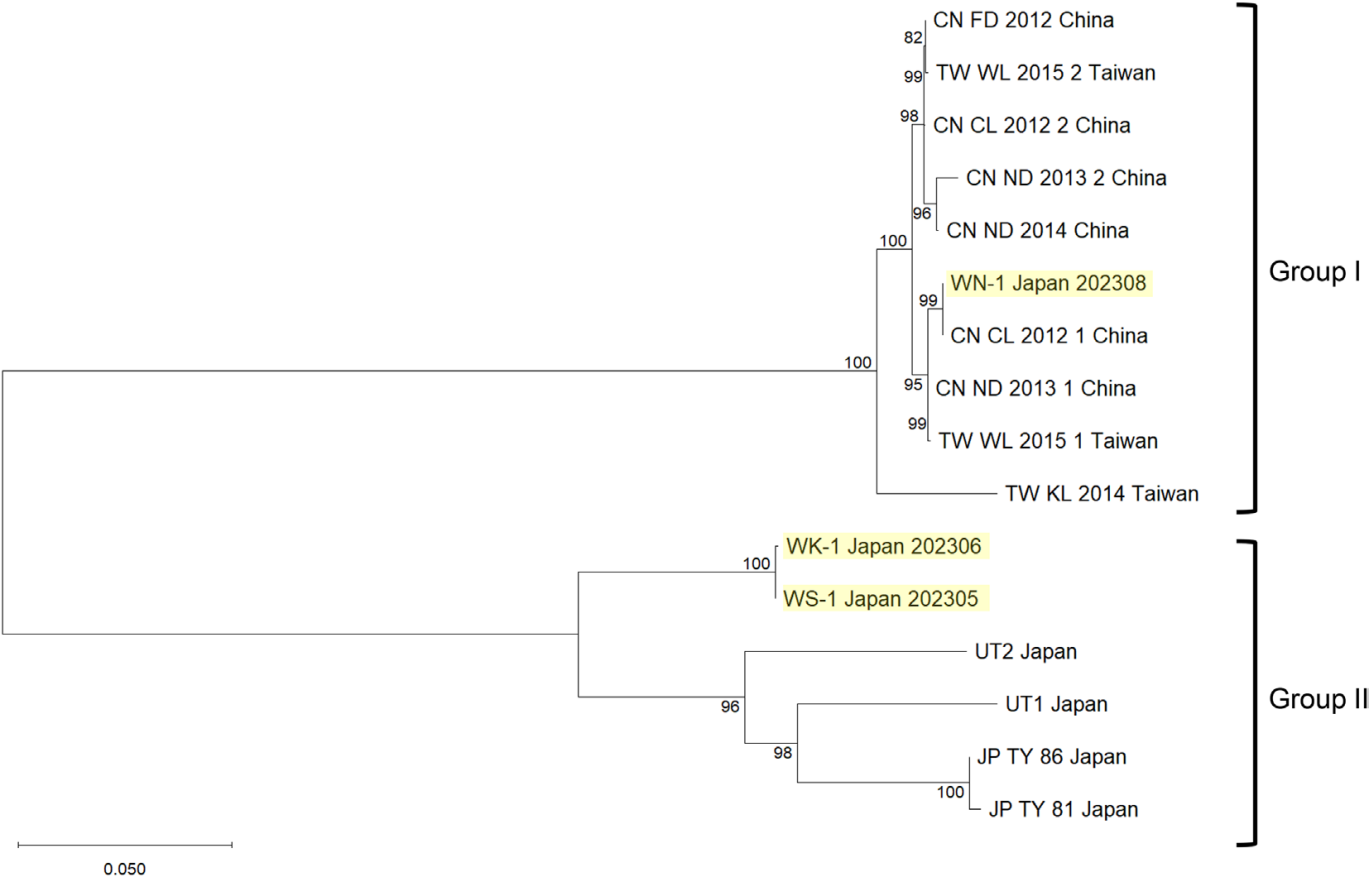
Maximum Likelihood (ML) phylogenetic tree of mitochondrial cytochrome c oxidase subunit 1 (cox‐1) gene sequences of *Cryptocaryon irritans*. Isolates from this study are highlighted in yellow. Bootstrap values of > 50% are indicated by branches (1000 replicates). The genetic grouping followed Chi et al. (2017).

**FIGURE 3:**
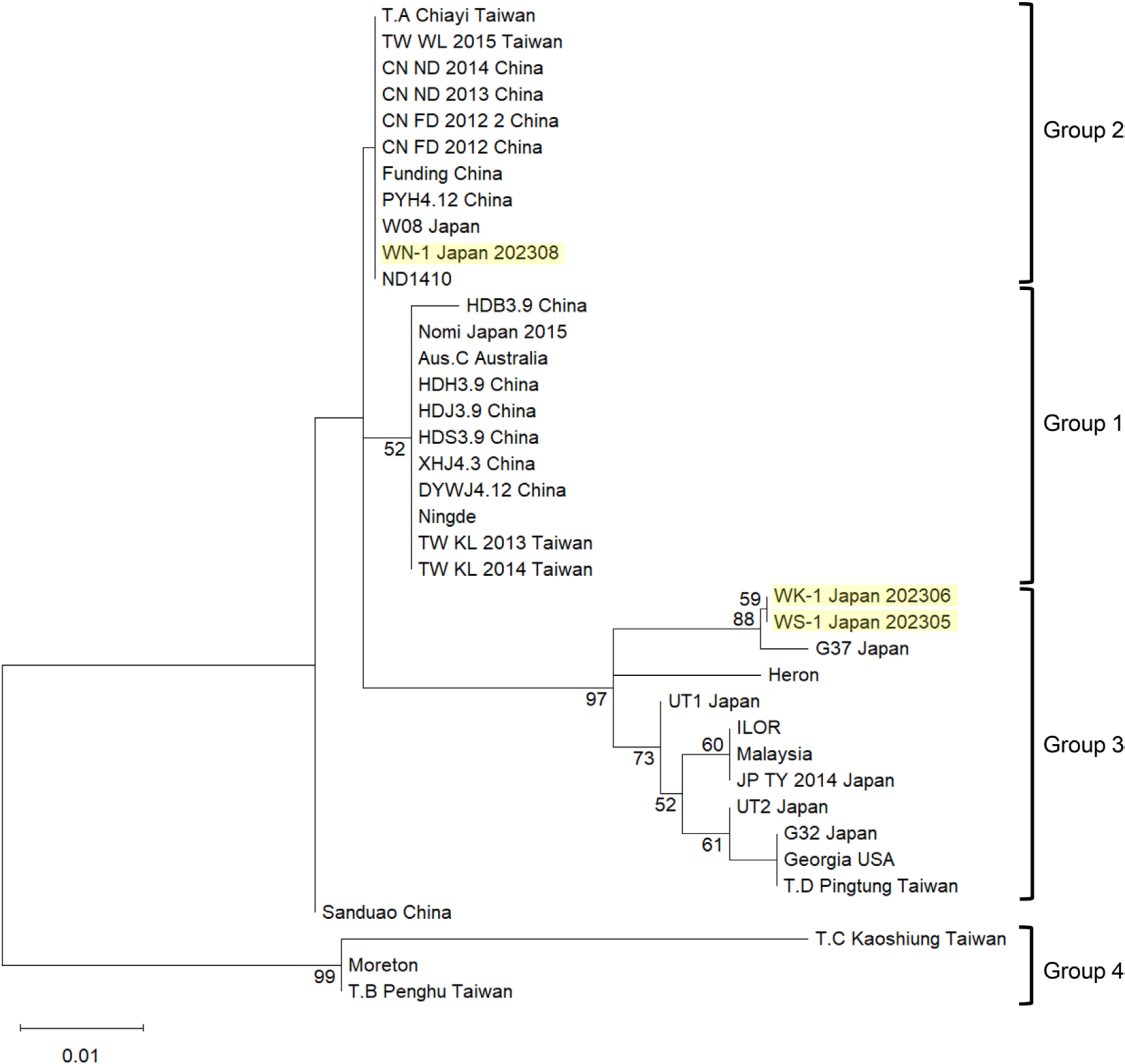
Maximum Likelihood (ML) phylogenetic tree of 18S rRNA, ITS‐1 sequences of *Cryptocaryon irritans*. Isolates from this study are highlighted in yellow. Bootstrap values of > 50% are indicated by branches (1000 replicates). The genetic grouping followed Sun et al. (2006).

Molecular phylogenetic analysis based on the nucleotide sequences of the mitochondrial DNA cytochrome c oxidase subunit 1 (cox‐1) region revealed that the WS‐1 (NCBI accession: LC896943) and WK‐1 (LC896944) isolates belonged to Group II, which consisted of isolates from Japan, as defined by Chi et al. (2017) (Figure 2). In contrast, the WN‐1 (LC896945) isolate was classified as Group I, which included isolates derived from China. Furthermore, phylogenetic analysis based on the nucleotide sequences of the 18S‐ITS1 region of the rRNA gene showed that the WS‐1 (LC896940) and WK‐1 (LC896941) isolates belonged to Group 3, which included isolates from Australia, Israel, Malaysia, Taiwan, the USA and Japan, whereas the WN‐1 (LC896942) isolate was placed in Group 2, which included isolates from China, Taiwan and Japan, as described by Sun et al. (2006) (Figure 3).

### 3.2 Measurement of Parasite Size

#### 3.2.1 Theront

**FIGURE 4:**
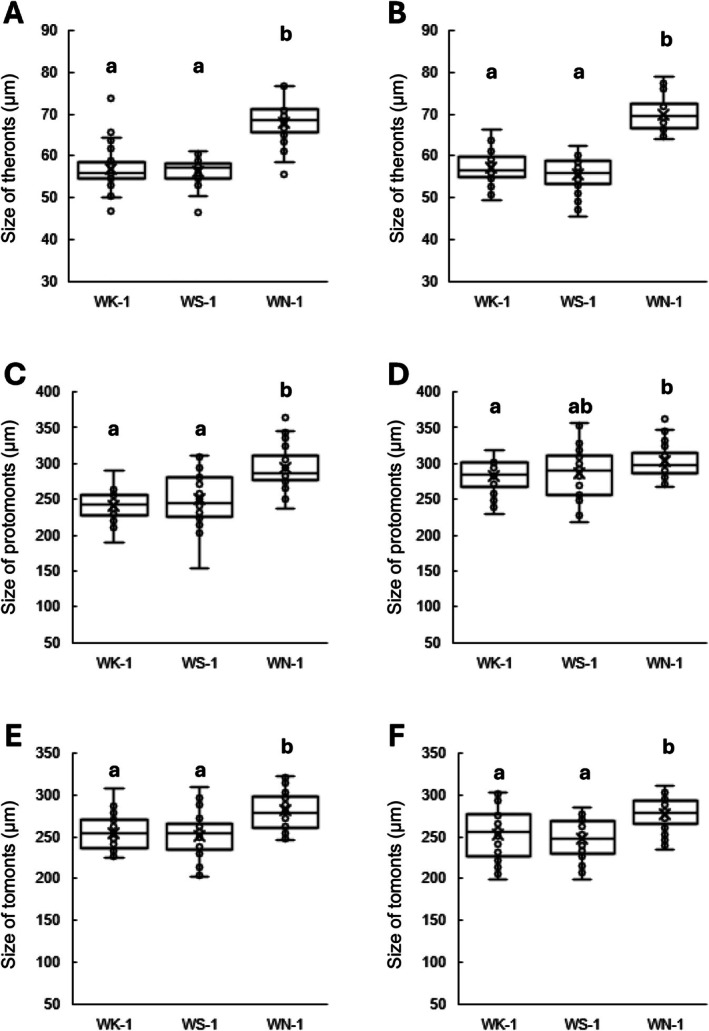
Size comparison at different developmental stages among isolates: Theronts (A, 1st trial; B, 2nd trial), Protomonts (C, 1st trial; D, 2nd trial) and Tomonts (E, 1st trial; F, 2nd trial). Different letters indicate significant differences (Steel‐dwass multiple comparison test, *p* < 0.05).

In the first measurement, the mean ± standard deviation (SD) values were as follows: WK‐1, 57.0 ± 5.4 μm; WS‐1, 56.3 ± 3.1 μm; and WN‐1, 68.0 ± 4.5 μm. There was no significant difference between WK‐1 and WS‐1 (*p* > 0.05); however, WN‐1 was significantly larger than the other two isolates (*p* < 0.05; Figure 4A). In the second measurement using parasites obtained on a different day, the sizes were: WK‐1, 57.1 ± 3.9 μm; WS‐1, 55.4 ± 4.2 μm; and WN‐1, 70.0 ± 3.9 μm. As in the first measurement, no significant difference was observed between WK‐1 and WS‐1 (*p* > 0.05). However, WN‐1 was significantly larger than the other two (*p* < 0.05; Figure 4B).

#### 3.2.2 Protomont

The mean major axis lengths of protomonts were as follows: WK‐1 isolate, 241.0 ± 20.1 μm; WS‐1, 247.5 ± 32.9 μm; and WN‐1, 295.8 ± 28.5 μm. No significant difference was found between WK‐1 and WS‐1 (*p* > 0.05), whereas that in WN‐1 was significantly larger (*p* < 0.05; Figure 4C). In the second measurement, the values were 281.9 ± 23.0 μm for WK‐1, 285.6 ± 35.0 μm for WS‐1 and 304.1 ± 25.9 μm for WN‐1. While no significant differences were observed between WK‐1 and WS‐1, or between WS‐1 and WN‐1 (*p* > 0.05), WN‐1 was significantly larger than WK‐1 (*p* < 0.05; Figure 4D).

#### 3.2.3 Tomont

The mean major axis lengths of tomonts were as follows: WK‐1, 254.7 ± 21.4 μm; WS‐1, 251.7 ± 26.4 μm; and WN‐1, 281.6 ± 22.3 μm. No significant differences were observed between WK‐1 and WS‐1 (*p* > 0.05), whereas that in WN‐1 was significantly larger (*p* < 0.05; Figure 4E). In the second measurement, the sizes were: WK‐1, 253.1 ± 30.0 μm; WS‐1, 247.0 ± 23.2 μm; and WN‐1, 277.8 ± 18.4 μm, with the same trend observed (*p* < 0.05; Figure 4F).

### 3.3 Number of Theronts Released From One Tomont

**FIGURE 5:**
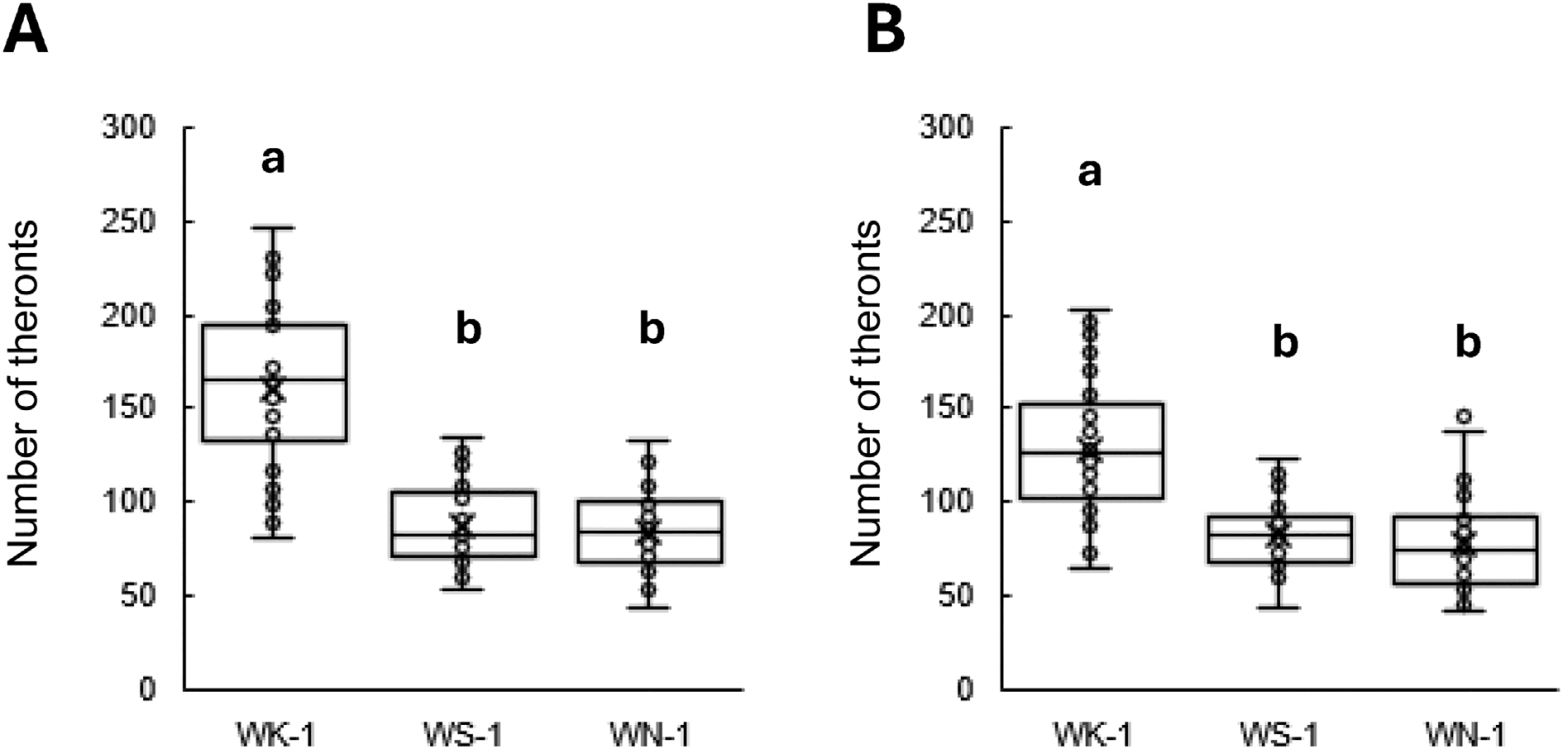
Comparison of theront production per tomont among isolates. (A) first trial; (B) second trial. Different letters indicate significant differences (Steel‐dwass multiple comparison test, *p* < 0.05).

In the first measurement, WK‐1 isolate released 160.8 ± 42.8 theronts, WS‐1 87.5 ± 22.1 theronts and WN‐1 83.5 ± 21.7 theronts. No significant difference was noted between the WS‐1 and WN‐1 isolates (*p* > 0.05); however, WK‐1 released significantly more theronts than the other two isolates (*p* < 0.05; Figure 5A). In the second measurement using tomonts obtained on a different day, similar results were obtained: WK‐1, 128.2 ± 35.8 theronts; WS‐1, 82.1 ± 18.6 theronts; and WN‐1, 77.6 ± 27.2 theronts. Again, WK‐1 released significantly more theronts than the other two isolates (*p* < 0.05; Figure 5B).

### 3.4 Immobilisation and Agglutination Assay

The results of the immobilisation and agglutination tests using antisera against each isolate are summarised in Table 3. When theronts of the WK‐1 isolate were incubated with antiserum raised against the isolate, an immobilisation reaction was observed (titre: 80). However, no immobilisation was observed when the same antiserum was reacted with theronts from the WS‐1 or WN‐1 isolates. Similarly, antisera raised against the WS‐1 and WN‐1 isolates only immobilised their respective homologous theronts (titre: 80), with no cross‐reactivity observed with other isolates.

**TABLE 3:** Results of immobilisation and agglutination assays.

Isolates	Antiserum elicited in fish against the *C. irritans*		
	WK‐1	WS‐1	WN‐1
WK‐1	80	< 20	< 20
WS‐1	< 20	80	< 20
WN‐1	< 20	< 20	80

*Note:* The table shows the maximum dilution titre of each antiserum at which immobilisation of theronts was observed. Values of < 20 indicate that no immobilisation or agglutination reactions were detected, even at a final dilution of 1:20.

### 3.5 Comparison of Pathogenicity

**FIGURE 6:**
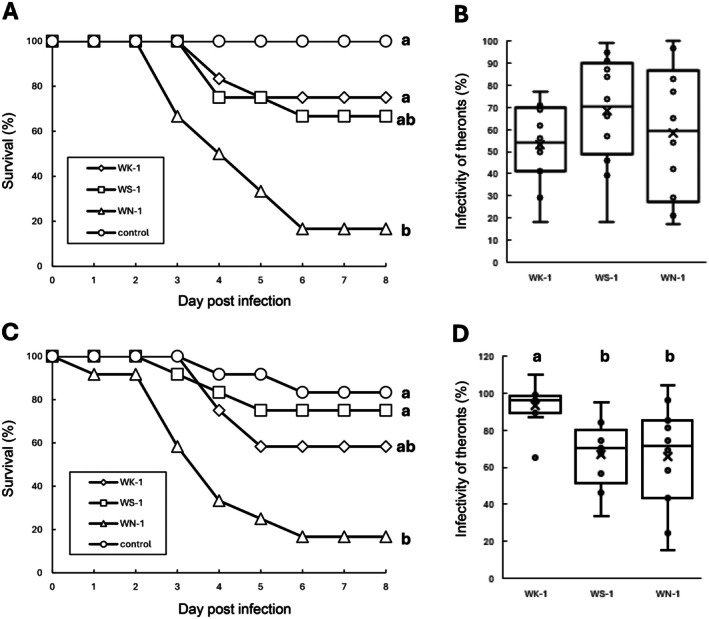
Survival and infectivity of black mollies following challenge with different isolates. (A) Survival rates in the first trial. (B) Infectivity in the first trial. (C) Survival rates in the second trial. (D) Infectivity in the second trial. Statistical differences were assessed using the log‐rank test, with *p*‐values adjusted by the Bonferroni correction for pairwise comparisons (A, C), and the Steel–Dwass multiple comparison test (B, D). Different letters indicate significant differences (*p* < 0.05). Different letters indicate significant differences (*p* < 0.05).

Seven days post‐infection, the survival rates of black mollies were as follows: WK‐1, 75%; WS‐1, 67%; WN‐1, 17%; and the negative control, 100%. While there were no significant differences between the WK‐1 and WS‐1 isolates or between the WS‐1 and WN‐1 isolates (*p* > 0.05), the survival rate in the WN‐1 group was significantly lower than that in the WK‐1 group (*p* < 0.05; Figure 6A). The infectivities of the theronts were as follows: in the first experiment: WK‐1, 53.0% ± 17.4%; WS‐1, 68.6% ± 23.8%; and WN‐1, 56.6% ± 16.7%, with no significant differences observed among the three isolates (*p* > 0.05; Figure 6B).

A similar trend was observed in the second experiment, with survival rates of 58% and 67% in the WS‐1, 17% for WN‐1 and 83% for the control group, respectively. In this trial, no significant differences were found between WK‐1 and WS‐1 or between WK‐1 and WN‐1 (*p* > 0.05); however, the WN‐1 isolate exhibited a significantly lower survival rate than the WS‐1 isolate (*p* < 0.05; Figure 6C). The infectivity of theront in the second experiment were: WK‐1, 90.7% ± 9.3%; WS‐1, 66.8% ± 18.0%; and WN‐1, 61.6% ± 25.2%. Although there was no significant difference between the WS‐1 and WN‐1 isolates (*p* > 0.05), the WK‐1 isolate had a significantly higher infection rate than the other two (*p* < 0.05; Figure 6D). No infection was observed in the control group.

**FIGURE 7:**
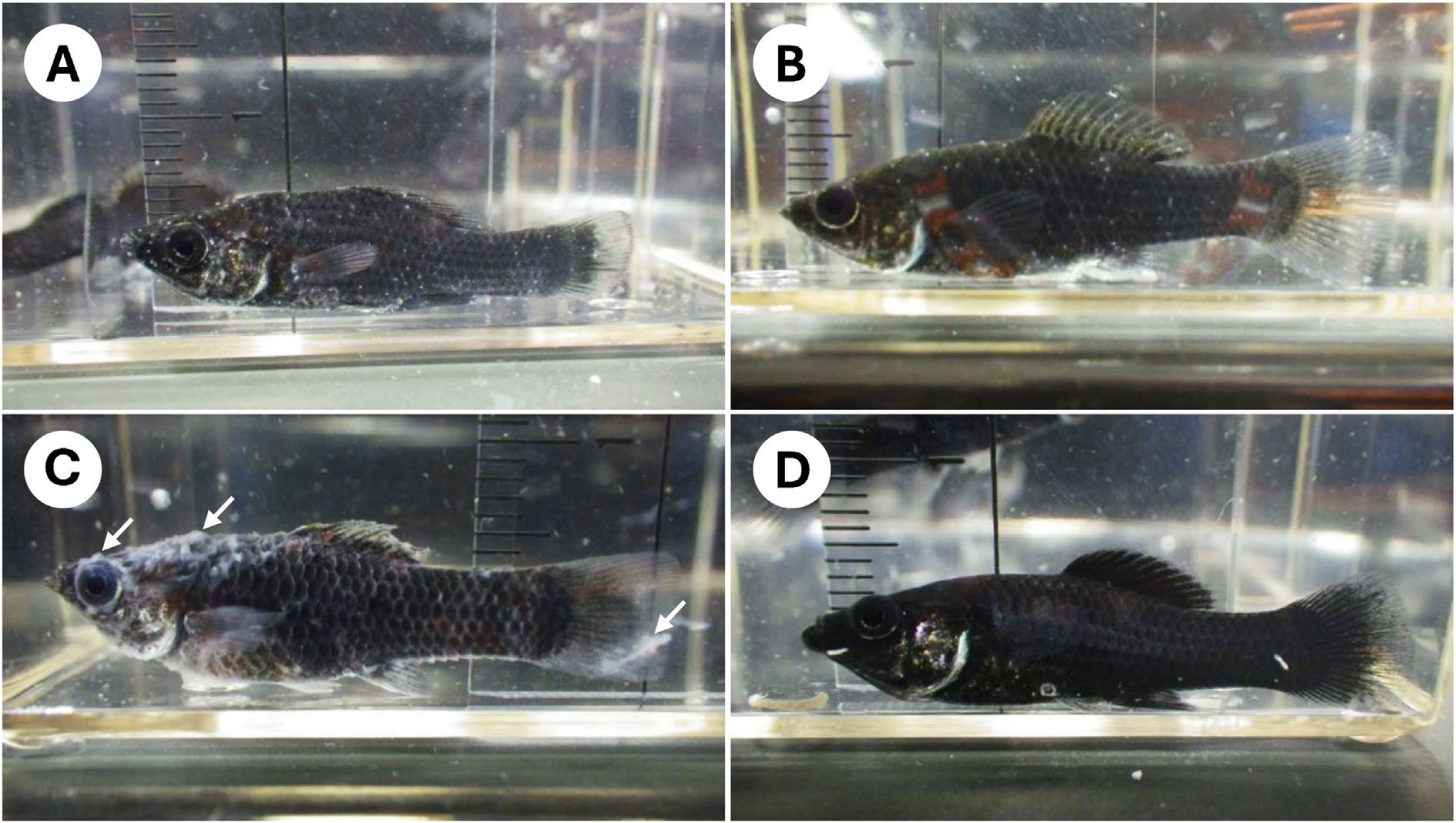
Infected fish at 2 days post‐infection with theronts. (A) Fish infected with the WK‐1 isolate. (B) Fish infected with the WS‐1 isolate. (C) Fish infected with the WN‐1 isolate. Excessive mucus secretion was observed on the body surface, fins and around the eyes; these areas are indicated by white arrows. (D) Uninfected fish.

Additionally, in both experiments, fish infected with the WN‐1 isolate displayed a tendency toward increased mucus production compared to those infected with other isolates (Figure 7).

**FIGURE 8:**
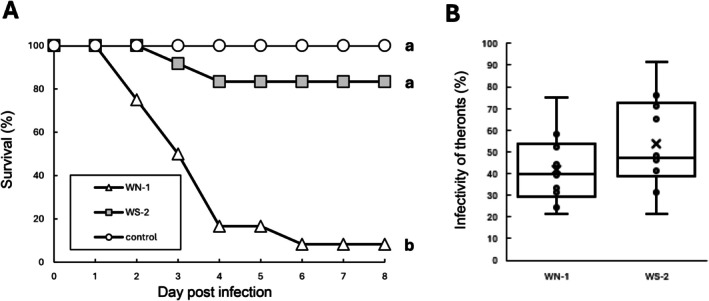
Survival rates of black mollies (A) and infection rates of theronts (B) in challenge experiments using the WN‐1 and WS‐2 isolates. Statistical differences were assessed using the log‐rank test, with *p*‐values adjusted by the Bonferroni correction for pairwise comparisons (A), and the Mann–Whitney U test (B). Different lowercase letters indicate significant differences in survival rates among groups (*p* < 0.05).

Furthermore, to evaluate the effect of passage duration on pathogenicity, black mollies were challenged with 1000 theronts per fish of either the WN‐1 or WS‐2 isolates. At 7 days post‐challenge, the survival rates of black mollies were 8.3% in the WN‐1 group, 83% in the WS‐2 isolate group and 100% in the control group. Survival in the WN‐1 isolate group was significantly lower than that in the WS‐2 group (*p* < 0.05; Figure 8A). In contrast, the infection rates per number of theronts inoculated were 41.7% ± 15.6% for the WN‐1 isolate and 53.6% ± 20.5% for the WS‐2 isolate, with no significant difference between the two groups (*p* > 0.05; Figure 8B). No infection was observed in the control group.

### 3.6 Salinity Conditions

#### 3.6.1 Cyst Formation of Protomonts Under Different Salinity Conditions

**FIGURE 9:**
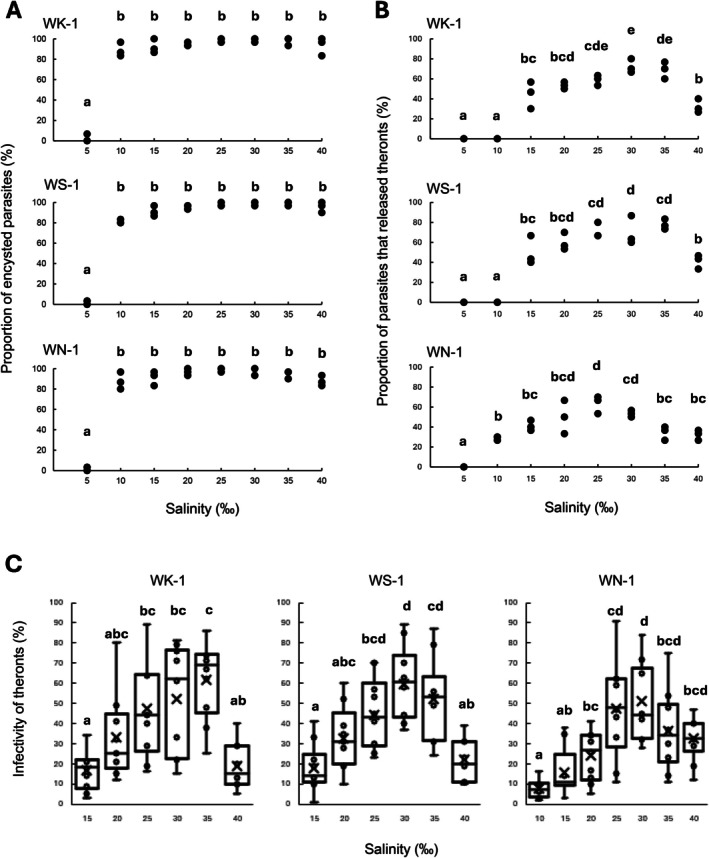
Effects of salinity on each isolate. (A) Proportion of parasites that formed cysts under different salinity conditions. (B) Proportion of parasites that released theronts under different salinity conditions. (C) Infectivity of theronts under different salinity conditions. Statistical differences were assessed using the Tukey–Kramer multiple comparison test after arcsine square root transformation of the data (A, B), and the Steel–Dwass multiple comparison test (C). Different letters indicate significant differences (*p* < 0.05).

The percentage of protomonts that formed cysts under salinity conditions ranging from 5‰ to 40‰ is shown in Figure 9A. At 5‰ salinity, the cyst formation rate was low (0%–7%) for all isolates and was significantly lower than that under other salinity conditions (*p* < 0.05). In contrast, at salinities of 10‰–40‰, more than 80% of protomonts from each isolate successfully encysted, with no significant differences observed among these salinity conditions (*p* > 0.05). The second experiment using protomonts collected on different days yielded similar results (Supporting Information Data S1, Figure S1A).

#### 3.6.2 Theront Release From Tomonts Under Different Salinity Conditions

Theront release rates under different salinity conditions are shown in Figure 9B. For the WK‐1 isolate, theront release remained above 50% at salinities from 20‰ to 35‰, with significantly higher rates observed at 30‰ and 35‰ compared to 15‰ and 40‰ (*p* < 0.05). For the WS‐1 isolate, release rates were also maintained above 50% at 15‰–35‰, with 30‰ showing significantly higher values than 15‰ and 40‰ (*p* < 0.05). For the WN‐1 isolate, release rates were above 50% at 20‰–30‰, but showed a tendency to decline at 15‰, 35‰ and 40‰. In addition, although not observed in the WK‐1 and WS‐1 isolates, the WN‐1 isolate released theronts even at 10‰, albeit at a low rate. The results of the second experiment are shown in Figure S1B. In the WK‐1 isolate, theront release remained above 55% at 20‰–35‰, but declined at 15‰ and 40‰. The WS‐1 isolate maintained a release rate above 50% at 20‰–40‰, but it declined at 15‰. The WN‐1 isolate maintained high release rates (> 55%) at 20‰–35‰, while 15‰ and 40‰ showed decreasing trends. Again, only the WN‐1 isolate showed theront release at 10‰.

#### 3.6.3 Infectivity of Theronts Under Different Salinity Conditions

The infectivity of the theronts under various salinity conditions is shown in Figure 9C. For the WK‐1 isolate, higher infection rates were observed at 25‰, 30‰ and 35‰. In contrast, infection rates dropped below 35% at 15‰–20‰ and 40‰. For the WS‐1 isolate, infection rates were maintained at high levels at 25‰, 30‰ and 35‰, but declined below 35% at 15‰–20‰ and 40‰. In the WN‐1 isolate, infection rates were high at 25‰ and 30‰, but decreased below 40% at 15‰–20‰ and 35‰–40‰. Notably, while no infection was observed at 10‰ in the WK‐1 and WS‐1 isolates, the WN‐1 isolate exhibited detectable infectivity even at 10‰, albeit at a low rate. The results of the second experiment are shown in Figure S1C. In the WK‐1 isolate, infection rates were maintained above 45% at 20‰–35‰, but were lower at 15‰ and 40‰. For the WS‐1 isolate, infection rates exceeded 45% at 25‰–35‰, while at 15‰–20‰ and 40‰, they dropped below 30%. In the WN‐1 isolate, high infection rates were observed at 30‰ and 35‰, whereas infection rates were below 40% at 15‰–25‰ and 40‰. As in the first experiment, only the WN‐1 isolate exhibited infectivity at 10‰.

### 3.7 Temperature Conditions

#### 3.7.1 Cyst Formation of Protomonts Under Different Temperature Conditions

**FIGURE 10:**
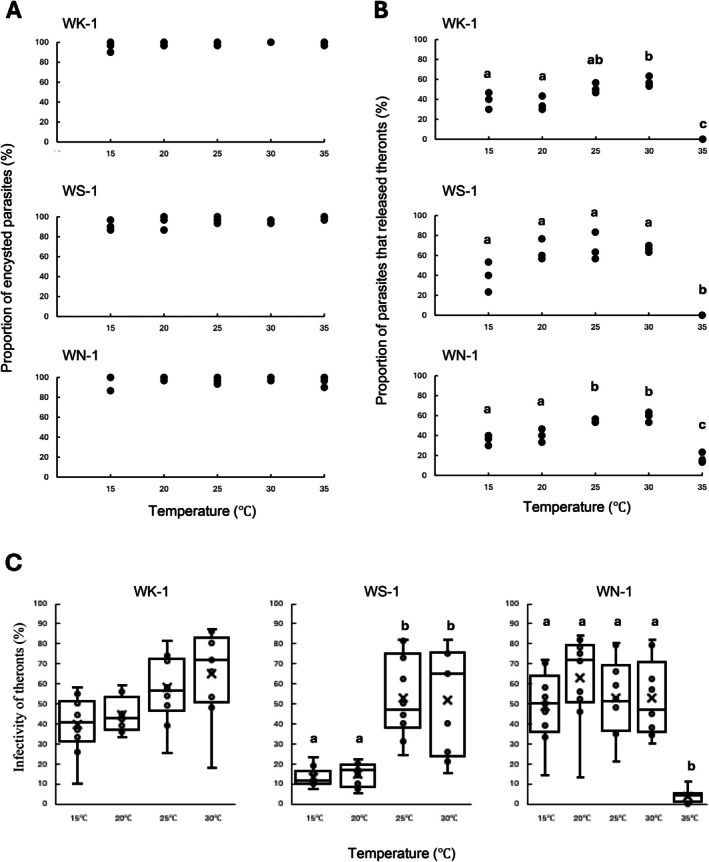
Effects of temperature on each isolate. (A) Proportion of parasites that formed cysts under different temperature conditions. (B) Proportion of parasites that released theronts under different temperature conditions. (C) Infectivity of theronts under different temperature conditions. Statistical differences were assessed using the Tukey–Kramer multiple comparison test after arcsine square root transformation of the data (A, B), and the Steel–Dwass multiple comparison test (C). Different letters indicate significant differences (*p* < 0.05).

In all isolates, over 85% of the protomonts formed cysts at all temperatures, and no significant differences were observed among the temperatures (*p* > 0.05; Figure 10A). In the second experiment using parasites collected on different days, cyst formation rates exceeded 80% in all isolates across all temperatures, with no significant differences detected (*p* > 0.05; Figure S2A).

#### 3.7.2 Theront Release From Tomonts Under Different Temperature Conditions

The release rates under different temperature conditions are shown in Figure 10B. In the WK‐1 isolate, higher release rates were observed at 25°C and 30°C, with the rate at 30°C being significantly higher than those at 15°C and 20°C (*p* < 0.05). Although no significant differences were found among temperatures in the WS‐1 isolate (*p* > 0.05), theront release tended to remain above 60% between 20°C and 30°C, while the rate at 15°C was relatively low. In the WN‐1 isolate, release rates were significantly higher at 25°C and 30°C than at 15°C and 20°C (*p* < 0.05). Additionally, theront release was observed at 35°C only in the WN‐1 isolate and not in the WK‐1 or WS‐1 isolates.

The results of the second experiment are presented in Figure S2B. The WK‐1 isolate showed high release rates at 25°C and 30°C, which were significantly higher than those at 15°C and 20°C (*p* < 0.05). For the WS‐1 isolate, rates were over 55% at 25°C and 30°C, with the 25°C rate being significantly higher than those at 15°C and 20°C (*p* < 0.05). In the WN‐1 isolate, release rates exceeded 65% at 25°C and 30°C, with the rate at 30°C significantly higher than those at 15°C and 20°C (*p* < 0.05). Notably, theront release at 35°C was again observed only in the WN‐1 isolate.

#### 3.7.3 Infectivity of Theronts Under Different Temperature Conditions

Theront release occurred 4 days after protomont collection at 30°C, 5 days at 25°C, 7 days at 20°C and 8 days at 15°C for all isolates. Accordingly, the challenge experiments were conducted as soon as sufficient theronts were available. For the WN‐1 isolate, theront release was also observed at 35°C on Day 4, and a challenge experiment was performed on that day. The results of the infection experiments are shown in Figure 10C. For the WK‐1 isolate, higher infection rates were observed at 25°C and 30°C, although these were not significantly different from the rates at 15°C and 20°C (*p* > 0.05). In contrast, the WS‐1 isolate exhibited significantly higher infection rates at 25°C and 30°C compared to 15°C and 20°C (*p* < 0.05). The WN‐1 isolate showed infection rates above 50% across 15°C–30°C with no significant differences among these temperatures (*p* > 0.05), but was also able to infect fish at 35°C, although at a low rate. The results of the second experiment are shown in Figure S2C. In the WK‐1 isolate, infection rates at 20°C, 25°C and 30°C were significantly higher than at 15°C (*p* < 0.05). For the WS‐1 isolate, infection rates were significantly higher at 25°C and 30°C than at 15°C and 20°C (*p* < 0.05). The WN‐1 isolate maintained infection rates above 45% across 15°C–30°C, with no significant differences among these conditions (*p* > 0.05), and again showed infectivity at 35°C, albeit at a low rate.

## 4 Discussion

Previous studies have shown that *C. irritans* isolates from different regions exhibit considerable variation in biological characteristics (Diggles and Lester 1996a; Yambot et al. 2003; Misumi et al. 2011). However, relatively little attention has been paid to the biological diversity of domestic isolates in Japan. Therefore, it remains unclear whether biologically distinct isolates of *C. irritans* exist in Japanese waters.

In this study, we established monoclonal isolates of *C. irritans* from three marine locations in the Wakayama Prefecture, Japan, and compared their biological characteristics to investigate the diversity of this parasite in geographically proximate coastal waters. Our results revealed differences among the isolates. The WN‐1 isolate was significantly larger than the other two at all developmental stages, whereas the WK‐1 isolate released significantly more theronts per tomont. These findings indicate inter‐isolate differences in size and reproductive output. Furthermore, the three isolates showed no cross‐reactivity in immobilisation assays, confirming that each represented a distinct serotype. Differences in the salinity and temperature ranges required for development and infection were also observed. Notably, the WN‐1 isolate remained infective even at low salinity (10‰) and high temperature (35°C) and exhibited an optimal temperature range distinct from that of the other isolates. Additionally, the survival rate of black mollies infected with the WN‐1 isolate was significantly lower than that of the other two isolates, suggesting a higher virulence of the WN‐1 isolate. Taken together, these results demonstrate that, even among parasites isolated from geographically close locations in Japan, there are clear biological differences, strongly suggesting the presence of genetically and phenotypically diverse isolates of *C. irritans* in Japan. To date, studies in Japan have rarely addressed the diversity of *C. irritans* , and the findings obtained from separate experiments using isolates of different origins have often been interpreted as common characteristics of the parasite. However, our findings suggest that domestic isolates exhibit distinct properties, indicating the need for future research to account for this diversity when designing experiments to gain insights into their ecological traits and developing control strategies.

Although isolate‐dependent variations in parasite size and number of theronts produced per tomont have been previously reported in overseas studies (Colorni 1985; Diggles and Lester 1996a; Yambot et al. 2003), the present study is the first to document such differences among isolates from Japan. These findings not only highlight the morphological and biological diversity among local *C. irritans* isolates but also have important implications for pathogenicity and disease spread. For instance, larger trophonts may cause more extensive physical damage to host tissues, potentially exacerbating the disease severity. Similarly, a higher theront production per tomont may accelerate the spread of infection and increase the risk of large‐scale outbreaks. The risk of cryptocaryoniasis outbreaks in Japan has been primarily attributed to aquaculture conditions. However, our findings suggest that the biological characteristics of *C. irritans* isolates in natural environments can influence the disease severity. Therefore, future studies should consider not only aquaculture conditions, but also the inherent properties of field isolates when assessing disease risk and impact.

A comparative analysis of temperature conditions revealed isolate‐dependent differences in infectivity. While previous studies reported optimal growth temperatures for *C. irritans* at 25°C–30°C, with marked inhibition of theront release and development at 34°C (Yoshinaga 1998) and delayed tomont maturation at 15°C (Kadohara 2013), our results demonstrated that the WN‐1 isolate remained infective even at 35°C and developed faster than previously reported at 15°C. These findings suggest that the thermal niche for disease development differs among the isolates, implying that the timing and severity of outbreaks in the field may be influenced by the temperature tolerance of local isolates. Therefore, predictive models and disease management strategies should incorporate the temperature responses of *C. irritans* present in the region.

Regarding salinity, both the WK‐1 and WS‐1 isolates caused infection at 15‰–40‰, while the WN‐1 isolate remained infective even at 10‰. This challenges earlier findings suggesting that *C. irritans* cannot develop properly below 20‰ and that low‐salinity treatments may be effective for disease control (Cheung et al. 1979; Colorni 1985). Our results indicate that some isolates can survive and cause disease even under low‐salinity conditions, suggesting that salinity‐based treatments should be reevaluated considering the diversity of isolates. On the other hand, the salinity range most suitable for infection (30‰–35‰) did not differ significantly among the isolates, suggesting that isolates with different biological traits can coexist in the same salinity environment. This possibility should be considered in future field surveys aimed at characterising strain composition in aquaculture and natural habitats.

The discovery that all three isolates used in this study belonged to different serotypes provides important insights into vaccine development against *C. irritans* . Previous studies have demonstrated that fish infected with *C. irritans* acquire strong protective immunity (Yoshinaga and Nakazoe 1997), which is induced by immobilisation antigens (i‐antigens) that define isolated serotypes (Hatanaka et al. 2007; Misumi et al. 2011). Based on these findings, vaccine development efforts have focused on i‐antigens, and studies have shown promising results in several countries (Priya et al. 2012; Huang et al. 2012; Lokanathan et al. 2016). However, the potential for serotype diversity has raised concerns regarding the efficacy of i‐antigen‐based vaccines (Misumi et al. 2011). Although such diversity has not yet been confirmed, our study provides direct evidence for serotype variation among geographically close isolates. Furthermore, immobilisation tests using antisera from this study failed to immobilise *C. irritans* newly isolated from the same locations, suggesting the existence of multiple serotypes, even within the same site (Supporting Information Data S2, Tables S3 and S4). These results imply that vaccines based on a single i‐antigen may not provide broad protection and that a new strategy capable of addressing serotype diversity is required for effective vaccine development.

Although the existence of isolates with differing pathogenicity has been suggested previously (Diggles and Lester 1996b), no experimental evidence has confirmed this to date. In our study, despite similar infection intensities across isolates, fish infected with the WN‐1 isolate exhibited significantly lower survival rates, confirming its higher pathogenicity. To exclude the possibility that this was due to differences in culture passage number (as prolonged maintenance is known to attenuate infectivity; Burgess and Matthews 1994), we compared it with a less‐passaged isolate, and the WN‐1 isolate still showed higher pathogenicity (Figure 8). Thus, we provided experimental evidence that *C. irritans* exhibits intraspecific variation in pathogenicity.

The cause of the higher pathogenicity observed in the WN‐1 isolate remains unclear, but its larger body size at all stages compared to other isolates may result in greater host tissue damage, contributing to increased pathogenicity. Notably, an unusual clinical feature, excessive mucus secretion, was observed in black mollies infected with the WN‐1 isolate, but not in fish infected with the other two isolates (Figure 7). Additionally, although previous studies have reported that fish mortality is typically associated with tissue damage caused by trophont detachment during maturation (Colorni and Burgess 1997; Pironet and Jones 2000), in the present study, fish infected with the WN‐1 isolate died before the parasites visibly matured on the skin surface. This suggests that a distinct pathological mechanism is involved in the pathogenicity of this isolate. However, as histopathological analyses and inflammatory marker evaluations were not conducted, this mechanistic interpretation may not be fully accurate. Therefore, further studies are needed to investigate the potential relationship between pathogenicity, increased parasite body size and enhanced mucus production. Additionally, since the pathogenicity comparisons in this study were limited to black mollies, future research should determine whether similar differences exist across other host species.

In the present study, the most pathogenic isolate (WN‐1) originated from a cultured fish, whereas the other two isolates were obtained from wild fish. Although the present study did not assess the effect of host origin, it is possible that differences in environmental conditions between aquaculture and natural systems contributed to the observed phenotypic divergence. Aquaculture environments are typically characterised by high host density, reduced genetic diversity of host populations and repeated introduction of naïve hosts, conditions that closely resemble serial‐passage scenarios wherein parasite transmission is less constrained by host mortality and opportunities for infection are frequent. Under such conditions, parasite fitness is expected to depend primarily on transmission efficiency rather than host survival, ay favouring the maintenance or emergence of highly pathogenic phenotypes (Nowak 2007). In addition, cultured fish are confined to limited space and cannot avoid infective stages of parasites, which may enhance transmission opportunities and weaken the influence of the trade‐off between pathogenicity and host survival observed in wild systems. Given the direct life cycle and rapid reinfection dynamics of *C. irritans* , such culture‐related selective pressures may be particularly relevant for this parasite. However, the number of isolates examined in this study was limited. Therefore, the higher pathogenicity of the WN‐1 isolate cannot be attributed solely to its cultured origin. Further studies using multiple isolates from both wild and cultured fish under comparable maintenance conditions will be required to disentangle the relative contributions of host origin, environmental conditions and laboratory passage to pathogenicity.

In addition, only a single clone was established and analysed per fish in this study. Genetically and phenotypically distinct clones may coexist within a single host, and such intra‐host diversity could influence estimates of virulence and environmental tolerance. Consequently, the present results represent inter‐isolate comparisons and may not fully reflect the range of variation within individual infections. Future studies analysing multiple clones per host will be necessary to evaluate within‐host diversity and its potential contribution to pathogenicity.

In this study, molecular phylogenetic analyses were conducted to clarify the genetic differences between the three isolates. Previous studies have shown that *C. irritans* can be classified into four genetic groups (1–4) based on the 18S‐ITS1 region and into two groups (I and II) based on the cox‐1 region (Sun et al. 2006; Chi et al. 2017). The analysis revealed that the WK‐1 and WS‐1 isolates belonged to group 3 for the 18S‐ITS1 region and group II for the cox‐1 region, whereas the WN‐1 isolates belonged to Groups 2 and 1, respectively. Thus, the WK‐1 and WS‐1 isolates were closely related, whereas the WN‐1 isolate was phylogenetically distinct. Previous studies have reported differences in the biological characteristics of distinct genetic groups (Diggles and Adlard 1997; Yambot et al. 2003; Sun et al. 2006). However, the present study demonstrated that differences in characteristics were observed not only between distantly related isolates such as WK‐1/WS‐1 and WN‐1, but also between closely related isolates such as WK‐1 and WS‐1. These findings indicate that the diversity of *C. irritans* extends beyond intergroup differences and may also occur within the same genetic group. Although the three isolates examined in this study were obtained from adjacent coastal areas, they exhibit distinct biological characteristics. Considering that cryptocaryoniasis outbreaks have been reported across many regions in Japan, it is likely that many genetically and biologically diverse isolates exist domestically. Therefore, in Japan, *C. irritans* should not be regarded as a uniform pathogen; instead, future ecological studies and the development of control measures must consider its diversity.

In conclusion, this study demonstrated substantial biological variation among three *C. irritans* isolates obtained from the neighbouring coastal regions of Wakayama Prefecture. Our findings indicated the presence of diverse *C. irritans* isolates with distinct characteristics in Japan. These insights are expected to contribute significantly to future research aimed at improving control strategies and developing predictive models for cryptocaryoniasis.

## Author Contributions

**Hiromi Matsuoka:** investigation, writing – original draft. **Toshihide Iwatsuki:** investigation. **Maho Kotake:** investigation. **Tomoki Honryo:** resources. **Sho Shirakashi:** sample collection, supervision. **Naoki Itoh:** supervision. **Yuho Watanabe:** conceptualization, investigation, writing – review and editing, supervision.

## Funding

This work was supported by Japan Society for the Promotion of Science KAKENHI (grant no. 24K17950).

## Conflicts of Interest

The authors declare no conflicts of interest.

## Supporting information


**Figure S1:** Effects of salinity on each isolate. (A) Proportion of parasites that formed cysts under different salinity conditions. (B) Proportion of parasites that released theronts under different salinity conditions. (C) Infectivity of theronts under different salinity conditions. Statistical differences were assessed using the Tukey–Kramer multiple comparison test after arcsine square root transformation of the data (A, B), and the Steel–Dwass multiple comparison test (C). Different letters indicate significant differences (*p* < 0.05).
**Figure S2:** Effects of temperature on each isolate. (A) Proportion of parasites that formed cysts under different temperature conditions. (B) Proportion of parasites that released theronts under different temperature conditions. (C) Infectivity of theronts under different temperature conditions. Statistical differences were assessed using the Tukey–Kramer multiple comparison test after arcsine square root transformation of the data (A, B), and the Steel–Dwass multiple comparison test (C). Different letters indicate significant differences (*p* < 0.05).
**Table S1:**. Information on samples used for phylogenetic analysis of the Cox‐1 region.
**Table S2:**. Information on samples used for phylogenetic analysis of the 18S‐ITS1 region.
**Table S3:** Information of additional samples of *Cryptocaryon irritans*.
**Table S4:** Results of immobilisation and agglutination assays using additional isolates. The table shows the maximum dilution titre of each antiserum at which immobilisation of theronts was observed. Values of < 20 indicate that no immobilisation or agglutination reaction was detected even at the final dilution of 1:20. For the newly obtained isolates WS‐3 and WN‐2, long‐term maintenance of parasites and preparation of homologous antisera were not possible due to space limitations. Therefore, only heterologous antisera from other isolates were tested to determine whether they shared the same serotype with previously established isolates. These supplementary experiments were conducted on a separate occasion

## Data Availability

The data that support the findings of this study are available from the corresponding author upon reasonable request.
