# Supplementary material for: Intraspecific Variation in the Characteristics of Cryptocaryon irritans Isolated in Japan
Source: J Fish Dis. 2026 Apr 1;49(9):e70177. doi: 10.1111/jfd.70177 (PMC13431706; doi:10.1111/jfd.70177)
Supplement: Supplementary file 1 — Figure S1: Effects of salinity on each isolate. (A) Proportion of parasites that formed cysts under different salinity conditions. (B) Proportion of parasites that released theronts under different salinity conditions. (C) Infectivity of theronts under different salinity conditions. Statistical differences were assessed using the Tukey–Kramer multiple comparison test after arcsine square root transformation of the data (A, B), and the Steel–Dwass multiple comparison test (C). Different letters indicate significant differences (p < 0.05). Figure S2: Effects of temperature on each isolate. (A) Proportion of parasites that formed cysts under different temperature conditions. (B) Proportion of parasites that released theronts under different temperature conditions. (C) Infectivity of theronts under different temperature conditions. Statistical differences were assessed using the Tukey–Kramer multiple comparison test after arcsine square root transformation of the data (A, B), and the Steel–Dwass multiple comparison test (C). Different letters indicate significant differences (p < 0.05). Table S1:. Information on samples used for phylogenetic analysis of the Cox‐1 region. Table S2:. Information on samples used for phylogenetic analysis of the 18S‐ITS1 region. Table S3: Information of additional samples of Cryptocaryon irritans. Table S4: Results of immobilisation and agglutination assays using additional isolates. The table shows the maximum dilution titre of each antiserum at which immobilisation of theronts was observed. Values of < 20 indicate that no immobilisation or agglutination reaction was detected even at the final dilution of 1:20. For the newly obtained isolates WS‐3 and WN‐2, long‐term maintenance of parasites and preparation of homologous antisera were not possible due to space limitations. Therefore, only heterologous antisera from other isolates were tested to determine whether they shared the same serotype with previously established isolates. These supp [file JFD-49-e70177-s001.zip › jfd70177-sup-0001-TableS1-S2-FigureS1-S2.pdf]

Table S1. Information on samples used for phylogenetic analysis of the Cox-1 region.

Samples	Locations	Accession Numbers
CN_FD_2012	China	KY426994
CN_CL_2012_1	China	KY426995
CN_CL_2012_2	China	KY426996
CN_ND_2013_1	China	KY426997
CN_ND_2013_2	China	KY426998
CN_ND_2014	China	KY426999
TW_KL_2014	Taiwan	KY427000
TW_WL_2015_1	Taiwan	KY427001
TW_WL_2015_2	Taiwan	KY427002
JP_TY_81	Japan	KY427003
JP_TY_86	Japan	KY427004
UT1	Japan	LC627770
UT2	Japan	LC627771

Table S2. Information on samples used for phylogenetic analysis of the 18S-ITS1 region.

Samples	Locations	Accession Numbers
G32	Japan	AB381933
G37	Japan	AB381934
W08	Japan	AB608054
T.A_Chiayi	Taiwan	AF490381
T.B_Penghu	Taiwan	AF490382
T.C_Kaoshiung	Taiwan	AF490383
T.D_Pingtung	Taiwan	AF490384
Malaysia	Malaysia	AF490385
ILOR Elat	Israel	AY029269
Georgia	USA	AY029270
Aus.A_Heron island	Australia	AY029271
Aus.B_Moreton Bay	Australia	AY029272
Aus.C	Australia	AY029273
HDB3.9	China	DQ270008
HDH3.9	China	DQ270009
PYH4.12	China	DQ270010
HDJ3.9	China	DQ270011
DYWJ4.12	China	DQ270012
XHJ4.3	China	DQ270013
HDS3.9	China	DQ270014
Fuding	China	HQ148713
Sanduo	China	HQ148714
Ningde	China	KC357673
ND1410	China	KU761582
CN_FD_2012	China	KY486177
CN_CL_2012	China	KY486178
CN_ND_2013	China	KY486179
CN_ND_2014	China	KY486180
JP_TY_2014	Japan	KY486181
TW_KL_2013	Taiwan	KY486182
TW_KL_2014	Taiwan	KY486183
TW_WL_2015	Taiwan	KY486184
Nomi	Japan	LC128576
UT1	Japan	LC627766
UT2	Japan	LC627767

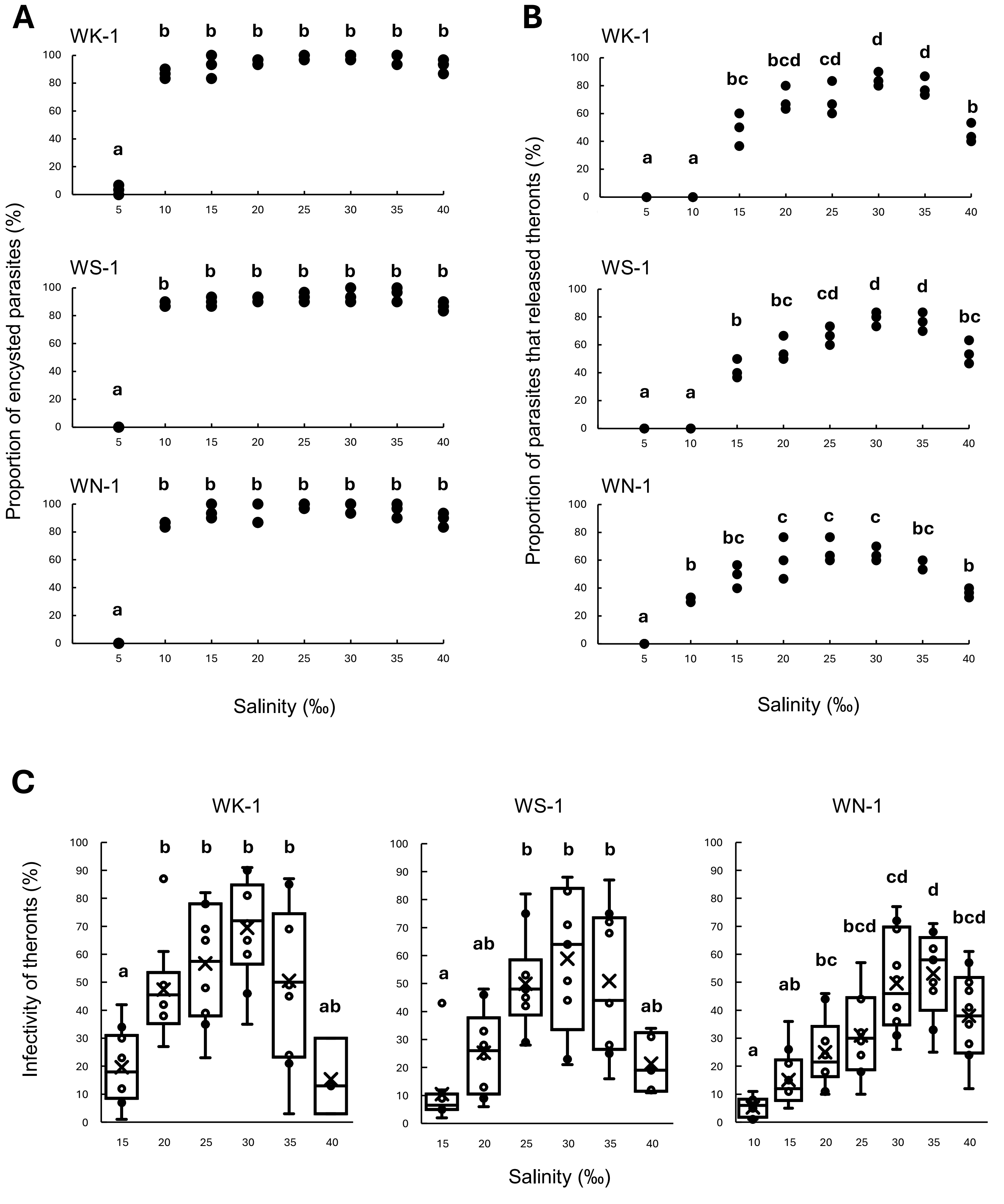

Fig. S1. Effects of salinity on each isolate. (A) Proportion of parasites that formed cysts under different salinity conditions. (B) Proportion of parasites that released theronts under different salinity conditions. (C) Infectivity of theronts under different salinity conditions. Statistical differences were assessed using the Tukey–Kramer multiple comparison test after arcsine square-root transformation of the data (A, B), and the Steel–Dwass multiple comparison test (C). Different letters indicate significant differences ($P < 0.05$).

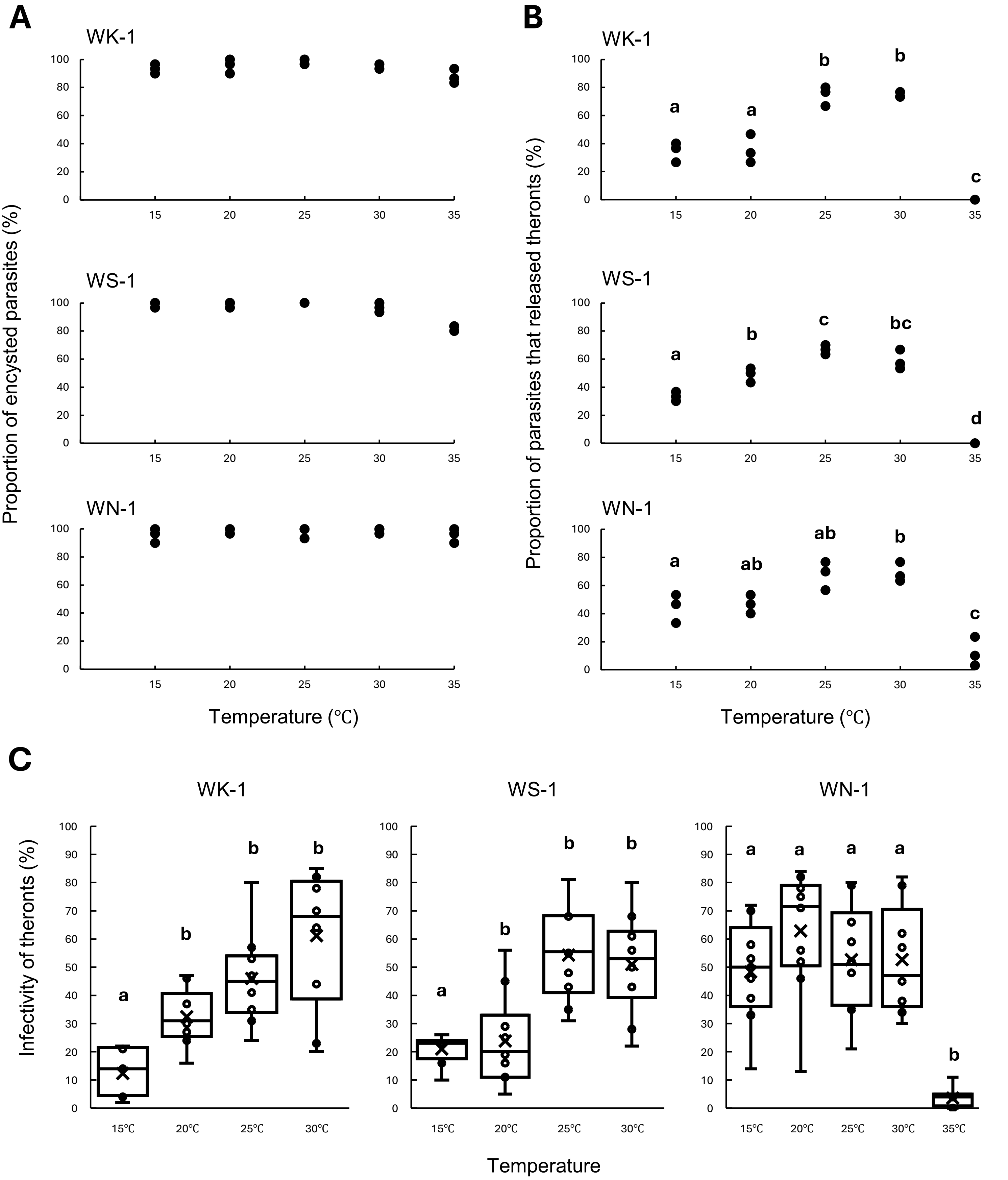

Fig. S2. Effects of temperature on each isolate. (A) Proportion of parasites that formed cysts under different temperature conditions. (B) Proportion of parasites that released theronts under different temperature conditions. (C) Infectivity of theronts under different temperature conditions. Statistical differences were assessed using the Tukey–Kramer multiple comparison test after arcsine square-root transformation of the data (A, B), and the Steel–Dwass multiple comparison test (C). Different letters indicate significant differences ($P < 0.05$).
