# Supplementary material for: Intraspecific Variation in the Characteristics of Cryptocaryon irritans Isolated in Japan
Source: J Fish Dis. 2026 Apr 1;49(9):e70177. doi: 10.1111/jfd.70177 (PMC13431706; doi:10.1111/jfd.70177)
Supplement: Supplementary file 1 — Figure S1: Effects of salinity on each isolate. (A) Proportion of parasites that formed cysts under different salinity conditions. (B) Proportion of parasites that released theronts under different salinity conditions. (C) Infectivity of theronts under different salinity conditions. Statistical differences were assessed using the Tukey–Kramer multiple comparison test after arcsine square root transformation of the data (A, B), and the Steel–Dwass multiple comparison test (C). Different letters indicate significant differences (p < 0.05). Figure S2: Effects of temperature on each isolate. (A) Proportion of parasites that formed cysts under different temperature conditions. (B) Proportion of parasites that released theronts under different temperature conditions. (C) Infectivity of theronts under different temperature conditions. Statistical differences were assessed using the Tukey–Kramer multiple comparison test after arcsine square root transformation of the data (A, B), and the Steel–Dwass multiple comparison test (C). Different letters indicate significant differences (p < 0.05). Table S1:. Information on samples used for phylogenetic analysis of the Cox‐1 region. Table S2:. Information on samples used for phylogenetic analysis of the 18S‐ITS1 region. Table S3: Information of additional samples of Cryptocaryon irritans. Table S4: Results of immobilisation and agglutination assays using additional isolates. The table shows the maximum dilution titre of each antiserum at which immobilisation of theronts was observed. Values of < 20 indicate that no immobilisation or agglutination reaction was detected even at the final dilution of 1:20. For the newly obtained isolates WS‐3 and WN‐2, long‐term maintenance of parasites and preparation of homologous antisera were not possible due to space limitations. Therefore, only heterologous antisera from other isolates were tested to determine whether they shared the same serotype with previously established isolates. These supp [file JFD-49-e70177-s001.zip › jfd70177-sup-0002-TableS3-S4.pdf]

Supplementary Data 2

Serotype comparation of additional *C. irritans* isolates obtained during the study period

Objective

In this study, we mainly compared the biological characteristics of three strains of *C. irritans*. However, several additional isolates were obtained during the study period. To complement the main experiments, we determined the serotypes of these isolates.

Materials and Methods

In addition to the three strains analyzed in the section 2.6 (WK-1, WS-1, and WN-1 isolates), two newly obtained isolates from Shirahama (WS-2 and WS-3) and one from Nachikatsuura (WN-2) were included. The origins of these isolates are summarized in Table S4. For serotype comparison, antisera were prepared against four isolates: WK-1, WS-1, WN-1 (as described in Section 2.6, using Japanese flounder), and WS-2. Immobilization and agglutination assays were conducted following the procedure described in Section 2.6.

Results

The results of serotype comparison by immobilization and agglutination assays are shown in Tables S5. When theronts of the WS-2 isolate were tested with anti-WS-2 serum, immobilization was observed (immobilization titer: 80). However, the same antiserum did not immobilize theronts of the WS-1, WK-1, or WN-1 isolates. Similarly, anti-WS-1 serum did not immobilize WS-2 theronts. Anti-WN-1 serum immobilized only WN-1 theronts (titer: 80) but showed no cross-reactivity with the WN-2 isolate or any other isolates.

Table S3. Information of additional samples of *C. irritans*.

Samples	Locations	Dates	Host	Sources
WS-2	Wakayama Pref. Shirahama	Mar. 2024	*Siganus fuscescens*	land-based facility
WS-3	Wakayama Pref. Shirahama	Jun. 2024	*Oplegnathus fasciatus*	land-based facility
WN-2	Wakayama Pref. Nachikatsuura	Nov. 2024	*Pagrus major*	Offshore fish farm

Table S4. Results of immobilization and agglutination assays using additional isolates.

The table shows the maximum dilution titer of each antiserum at which immobilization of theronts was observed. Values of <20 indicate that no immobilization or agglutination reaction was detected even at the final dilution of 1:20. For the newly obtained isolates WS-3 and WN-2, long-term maintenance of parasites and preparation of homologous antisera were not possible due to space limitations. Therefore, only heterologous antisera from other isolates were tested to determine whether they shared the same serotype with previously established isolates. These supplementary experiments were conducted on a separate occasion.

Isolates	Antiserum elicited in fish against the *C. irritans*			
	WK-1	WS-1	WN-1	WS-2
WK-1	80	<20	<20	<20
WS-1	<20	80	<20	<20
WN-1	<20	<20	80	<20
WS-2	<20	<20	<20	80
WS-3	<20	<20	<20	<20

Isolates	Antiserum elicited in fish against the *C. irritans*			
	WK-1	WS-1	WN-1	WS-2
WK-1	80	<20	<20	<20
WS-1	<20	80	<20	<20
WN-1	<20	<20	80	<20
WS-2	<20	<20	<20	80
WN-2	<20	<20	<20	<20
